# Associations of procalcitonin, C-reaction protein and neutrophil-to-lymphocyte ratio with mortality in hospitalized COVID-19 patients in China

**DOI:** 10.1038/s41598-020-72164-7

**Published:** 2020-09-14

**Authors:** Jian-bo Xu, Chao Xu, Ru-bing Zhang, Meng Wu, Chang-kun Pan, Xiu-jie Li, Qian Wang, Fang-fang Zeng, Sui Zhu

**Affiliations:** 1 Department of Critical Care Medicine, School of Clinical Medicine, Jiamusi University, No. 348, Dexiang Street, Jiamusi, 154000 Heilongjiang Province China; 2 Department of Neurology and Respirator Intensive Care Unit, Heilongjiang Provincial Hospital, No. 82, Zhongshan Road, Harbin, 150036 Heilongjiang Province China; 3 Department of Cardiology, School of Clinical Medicine, Jiamusi University, No. 348, Dexiang Street, Jiamusi, 154000 Heilongjiang Province China; 4 Department of Critical Care Medicine, Jiamusi Tumor Hospital, No. 37, Guanghua Road, Jiamusi, 154000 Heilongjiang Province China; 5 Department of Respiratory Medicine, Jiamusi Tumor Hospital, No. 37, Guanghua Road, Jiamusi, 154000 Heilongjiang Province China; 6 Department of Medical Statistics, School of Medicine, Jinan University, No. 601 Huangpu Road West, Guangzhou, 510632 Guangdong Province China; 7 Department of Epidemiology, School of Medicine, Jinan University, No. 601 Huangpu Road West, Guangzhou, 510632 Guangdong Province China

**Keywords:** Infectious diseases, Biomarkers, Prognostic markers

## Abstract

Coronavirus disease 2019 (COVID-19) is an important and urgent threat to global health. Inflammation factors are important for COVID-19 mortality, and we aim to explore whether the baseline levels of procalcitonin (PCT), C-reaction protein (CRP) and neutrophil-to-lymphocyte ratio (NLR) are associated with an increased risk of mortality in patients with COVID-19. A retrospective study was conducted and a total of 76 patients with confirmed COVID-19 were included between January 17, 2020 to March 2, 2020, of these cases, 17 patients were dead. After adjusting covariates, PCT (≥ 0.10 ng/mL) and CRP (≥ 52.14 mg/L) exhibited independent increasing risks of mortality were used hazard ratio (HR) of 52.68 (95% confidence interval *[CI]*: 1.77–1571.66) and 5.47 (95% *CI*: 1.04–28.72), respectively. However, NRL (≥ 3.59) was not found to be an independent risk factor for death in our study. Furthermore, the elevated PCT levels were still associated with increasing risk of mortality in the old age group (age ≥ 60 y), and in the critically severe and severe patients after adjustment for complications. Thu Baseline levels of PCT and CRP have been addressed as independent predictors of mortality in patients with COVID-19.

## Introduction

The mysterious outbreak of atypical pneumonia was reported in late December, 2019, in Wuhan, China, and the pathogen and its genomic characterization was quickly identified^1–3^. The highly infectious severe acute espiratorysyndromecoronavirus2 (SARS-CoV-2) began to spread to other countries around the world, which presented an important and urgent threat to global health. Then World Health Organization (WHO) named the disease as “Coronavirus disease 2019” (COVID-19) and declared it as a public health emergency of international concern on January 30, 2020. Globally, almost 2.5 million cases of COVID-19 and more than 160,000 deaths have been reported^4^, and a total of 82,798 confirmed cases with 4,632 deaths were reported in China up to April 22, 2020^5^.

Currently, no specific medicine has been developed against for this highly infectious disease. Although most patients have mild symptoms and good prognosis, patients with critically severe disease are at high risk of mortality. Therefore, it is urgent to find appropriate indicators to discriminate the severity and improve the progress to reduce the mortality rate of patients with COVID-19.

At present, several clinical features to estimate the risk of patients being experiencing a poor outcome from the infection could assist medical staff in triaging patients when allocating limited healthcare resources. A series of inflammation factors have been recognized as an enabling characteristic for COVID-19 severity and mortality^6–8^. As markers of systemic inflammation, procalcitonin (PCT), C-reaction protein (CRP) and neutrophil-to-lymphocyte ratio (NLR) are objective and easy to implement, and usually taken to measure patient’s susceptibility to mortality.

Li had found elevated serum CRP levels in the severe COVID-19 patients compared with the moderate patients^8^, however, from a cohort of 17 patients with COVID-19, Zhou did not found this situation in the aggravation group^9^. A retrospective study observed higher serum PCT and CRP levels in critical cardiovascular disease patients with COVID-19 than in the general patients^10^. A meta-analysis had shown that the NLR levels in severe patients with COVID-19 were increased compared with patients without severe disease^11^. Meanwhile, according to a retrospective study in early patients in Wuhan, Liu indicated NLR as an independent risk factor for mortality in hospitalized patients with COVID-19^12^.

Many studies were focused on the epidemic center of Wuhan, and the clinical characteristics and outcomes in other areas of Hubei province were not systematically reported. Xiaogan city is adjacent to the Wuhan, only 50 miles away, and the reported patients with COVID-19 was 3,518 up to April 24, 2020, ranked second in the Hubei province^13^. Hanchuan, one of seven county cities of Xiaogan, has a total area of 1658.56 km^2^ with the population of 1.08 million at the end of 2018^14^, and 763 cases with 29 cases dead were reported up to April 24, 2020^15^.

Due to controversial results and small sample size, there is still scarce and limited information regarding the inflammation factors associated with mortality in patients with COVID-19, especially for PCT, CRP and NLR. The relationship between these three factors and their independent associations with survival are not well studied. In this study, we aim to explore whether the baseline inflammation factors of PCT, CRP and NLR are associated with an increased risk of death in patients with COVID-19.

## Materials and methods

### Study design

This was a two-center, retrospective study in patients hospitalized to designated Hanchuan People’s Hospital and the First Hospital of Suihua. Suihua is small city located in the northeast of China, with a total of 47 confirmed cases up to April 24, 2020^16^. Because of small sample size, the critically severe patients from the First Hospital of Suihua were analyzed together. The informed consent were obtained from the patiens hospitalized to the above hospitals or theie relatives. This study was approved by the Institutional Ethics Board of Heilongjiang Provincial Hospital, and is in full compliance with the Helsinki Declaration.

### Study population

Patients with confirmed COVID-19 from January 17, 2020 to March 2, 2020 were enrolled in this study. All cases before hospitalization were laboratory-confirmed by human infection SARS-CoV-2 from throat swab samples^17,18^, following the recommendation by the National Institute for Viral Disease Control and Prevention of China^19^. According to the guidelines for diagnosis and management of COVID-19 (7th edition, in Chinese) issued by the National Health Commission of China, clinical conditions are classified into four types: mild, moderate, severe, and critically severe^20^. Because of patients with clinical mild type were in the mobile cabin hospitals, thus, in this research, we only enrolled patients of COVID-19 with moderate, severe and critically severe patients.

### Markers of inflammation

Laboratory measurements were conducted at hospitalization as close to initiation of antibiotics and antivirus as possible and received lab test when changing in health condition. Our primary measures of inflammation were PCT, CRP and NLR, because not all the patients were measured regularly, thus, we only included baseline levels of PCT, CRP and NRL during the treatment periods. The serum concentrations of PCT were determined using Maglumi 2000 Plus automated platform (Snibe, Shenzen, China) and CRP were measured by a specific protein immune analyzer using CRP-M100 instrument (Mindray, Shenzen, China). NLR was calculated for each case by dividing the reported total absolute neutrophil counts by the total absolute lymphocyte counts, which were detected by BC-6900 (Mindray, Shenzen, China) automatic blood cell analyzer.

### Other covariates

We reviewed the electronic medical records of patients with standardized data collection form to extract other covariates, including demographic (age, sex, time from illness onset to first hospital admission, chronic heart disease, chronic lung disease, chronic kidney disease, diabetes and hypertension), clinical symptoms (fever, cough, headache, myodynia, chills, nausea and vomiting, chest distress or shortness of breath), complications (acute respiratory distress syndrome [ARDS], hypohepatia, renal insufficiency, heart failure and shock) and therapeutic strategies including use of glucocorticoid, mechanical ventilation and immune globulin.

### Definition of clinical outcomes

The clinical outcomes, including discharge and death were monitored up to February 15, 2020 and March 19, 2020 in the First Hospital of Suihua and Hanchuan People’s Hospital, respectively. If the patient’s condition improved significantly and would be discharged when there were no respiratory symptoms, normal temperature for more than 3 days, and pulmonary imaging showed significant improvement in acute exudative lesions and most important thing was patients must pass 2 consecutive nucleic acid tests^20^.

### Statistics

Continuous variables were expressed as mean (standard deviation, SD) when values were normally distributed, otherwise, the results were presented in terms of median (interquartile range, IQR). The significant differences between the groups for continuous variables were compared using one-way analysis of variance when the data were normally distributed, otherwise, the Kruskal–Wallis H-test was used. Categorical variables were described as frequency and percentages, and proportions for categorical variables were compared using the independent sample chi-square test. The Logistic regression was used to prediction the combined effect.

To detect if PCT, CPR, NLR and their combined effect are more sensitive in predicting for mortality, the area under the curve (AUC) and the 95% *CI* of the receiver operator characteristic (ROC) curve was computed regarding discharge as negative whereas mortality as positive. The optimal cut‐off values for PCT, CPR, NLR and their combined effect to predict the mortality of patients with COVID‐19 were determined by Youden's index which could be utilized as the reference for clinical classification.

Survival curves of PCT, CPR, NLR and their combined effect were plotted using the Kaplan–Meier method and log-rank tests were used to compared difference. Cox proportional hazard ratio (*HR*) models were used to determine *HRs* and 95% *CIs* for each covariate to determine the risk factors for death during hospitalization in patients with COVID-19. Next, we examined PCT, CRP and NLR as independent predictors of survival in multivariable-adjusted Cox *HR* models, which were adjusted for age, sex, cancer, ARDS, hypohepatia, renal insufficiency, heart failure and shock. Furthermore, we also explored different effects stratified by age and sex, as well as clinical types. Because no death was in the moderate group, we only analyzed the risks of PCT, CPR and NLR in combined critically severe and severe groups. Time to death was defined as the time from hospital admission to mortality, and individuals alive were censored at the time of discharged.

Two-tailed* P* value less than 0.05 was considered statistically significant. All analyses were performed with R software, version 3.5.1.

## Results

### Characteristics among three groups

From January 29, 2020 to March 2, 2020, a total of 68 patients with COVID-19 from Hanchuan City people’s hospital and 8 cases from Suihua first hospital were included in this research, of these cases, 32 patients were in the critically severe group, 18 patients in the severe group, and the remaining 26 patients in the moderate group. The frequent clinical symptoms for patients with COVID-19 were fever (84.21%), cough (64.47%) and chest distress or shortness of breath (44.74%) on admission. Compared with moderate group, hypertension and chest distress or shortness of breath tended to be more common in the critically severe and severe groups (*P* < 0.05).

The patients with critical clinical type had higher rates of ARDS, renal insufficiency, heart failure ad shock compared with the other two groups, and renal insufficiency, heart failure and shock were only developed in critically severe group. More frequent treatments of glucocorticoid and mechanical ventilation were used in patients with critical severe and severe types than those with moderate clinical type.

22.37% (17/76) patients with COVID-19 were dead during the treatment, and those only developed in the critically severe group. Patients with moderate clinical type had longer days of stay in hospital (median 28 days) than in the critically severe (17 days) and severe groups (16.5 days), shown in Table 1.

**Table 1:** Characteristics of patients with COVID-19 in different clinical types in China.

Variables	Total (n = 76)	Critically severe group (n = 32)	Severe group (n = 18)	Moderate group (n = 26)	*P*
**Demographic**					
Age, mean (SD), years	59.11 (14.55)	59.12 (13.77)	60.72 (16.56)	57.96 (14.51)	0.830
Male	46 (60.53)	20 (62.5)	11 (61.11)	15 (57.69)	0.931
Time from illness onset to first hospital admission, median (IQR), days	7 (5, 13)	7 (5.25)	8.5 (9)	7 (7)	0.681
Chronic heart disease	7 (9.21)	2 (6.25)	2 (11.11)	3 (11.54)	0.748
Chronic lung disease	2 (2.63)	0 (0)	0 (0)	2 (7.69)	0.139
Chronic kidney disease	5 (6.58)	2 (6.25)	1 (5.56)	2 (7.69)	0.957
Diabetes	15 (19.74)	9 (28.12)	3 (16.67)	3 (11.54)	0.268
Hypertension	**27 (35.53**)	16 (50)*	8 (44.44)*	**3 (11.54)**	**0.006**
Cancer	3 (3.95)	2 (6.25)	1 (5.56)	0 (0)	0.441
**Clinical symptoms**					
Fever	64 (84.21)	27 (84.38)	14 (77.78)	23 (88.46)	0.633
Cough	49 (64.47)	22 (68.75)	12 (66.67)	15 (57.69)	0.665
Headache	2 (2.63)	1 (3.12)	0 (0)	1 (3.85)	0.717
Myodynia	2 (2.63)	2 (6.25)	0 (0)	0 (0)	0.244
Chills	8 (10.53)	4 (12.5)	2 (11.11)	2 (7.69)	0.835
Nausea and vomiting	2 (2.63)	2 (6.25)	0 (0)	0 (0)	0.244
Chest distress or shortness of breath	**34 (44.74)**	18 (56.25)*	11 (61.11)*	**5 (19.23)**	**0.005**
**Complications**					
ARDS	**25 (32.89)**	23 (71.88)*^#^	**2 (11.11)**	**0 (0)**	** < 0.001**
Hypohepatia	13 (17.11)	9 (28.12)	2 (11.11)	2 (7.69)	0.09
Renal insufficiency	**6 (7.89)**	6 (18.75)*^#^	**0 (0)**	**0 (0)**	**0.011**
Heart failure	**7 (9.21)**	7 (21.88)*^#^	**0 (0)**	**0 (0)**	**0.005**
shock	**15 (19.74)**	15 (46.88)*^#^	**0 (0)**	**0 (0)**	** < 0.001**
**Therapy and outcomes**					
Glucocorticoid	**59 (77.63)**	31 (96.88)*	15 (83.33)*	**13 (50)**	** < 0.001**
Mechanical ventilation	**52 (68.42)**	32 (100)*	18 (100)*	**2 (7.69)**	** < 0.001**
Immune globulin	**48 (63.16)**	29 (90.62)*^#^	**9 (50)**	**10 (38.46)**	** < 0.001**
Length of hospitalization, median (IQR), days	**18.5 (13.75, 29.25)**	17 (9)*	16.5 (7)*	**28 (15.75)**	**0.009**
All cause of death	**17 (22.37)**	17 (53.12)*^#^	**0 (0)**	**0 (0)**	** < 0.001**

Data in bold indicate values of statistical significance.

*SD* standard deviation, *IQR* interquartile range, *ARDS* acute respiratory distress syndrome.

Compared with moderate group, *P < 0.05; Compared with sever group, ^#^*P* < 0.05.

### Relationships between PCT, CRP, NLR and clinical types

**Figure 1:**
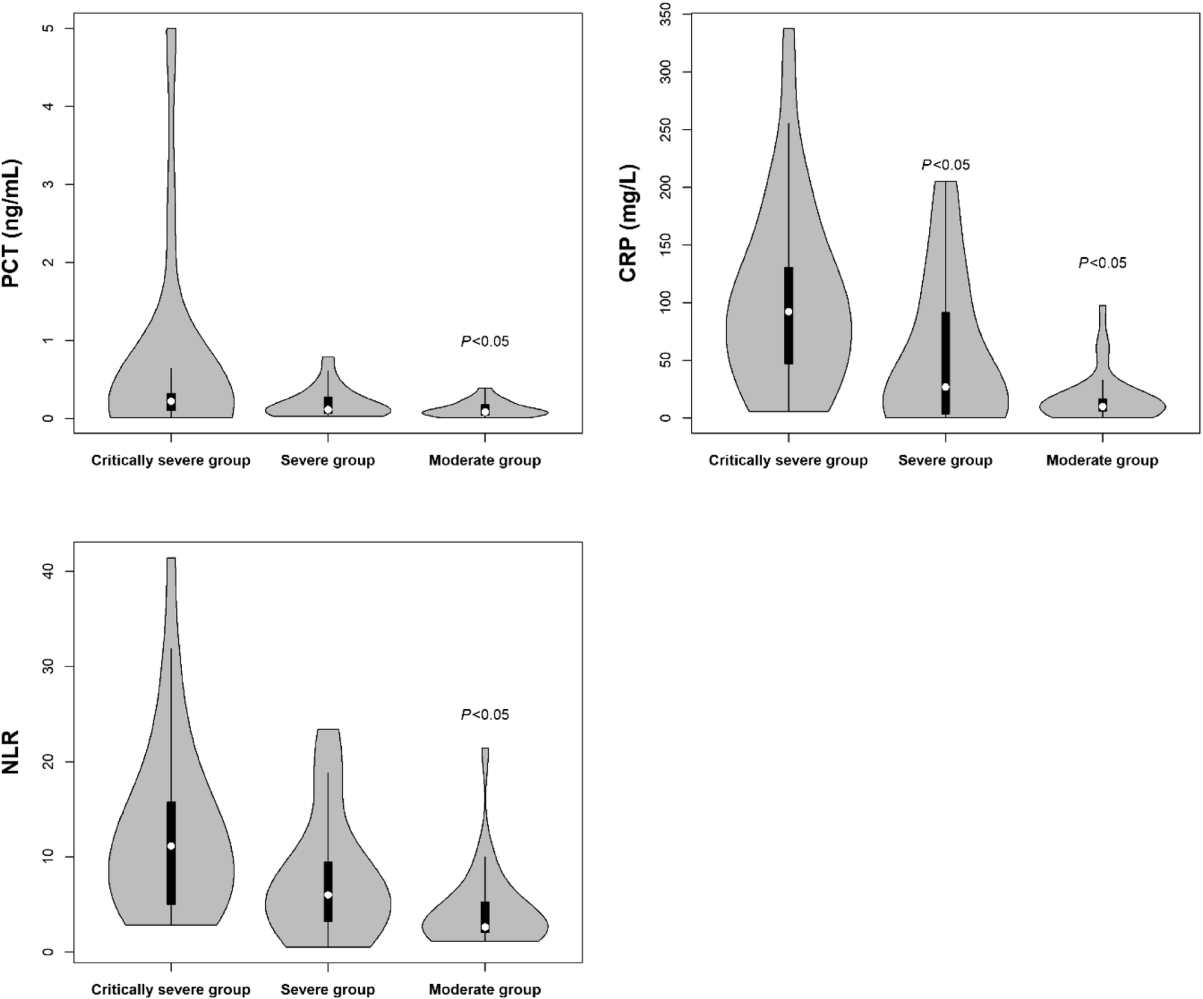
The violin plots of PCT, CRP and NLR levels in patients with COVID-19 in critically severe group, severe group and moderate group in China. The white dot in the violin was the median value of inflammatory factor, and the black rectangle was the percentile 25th and 75th.

The PCT, CRP and NLR displayed statistically significant among three groups, and patients with critical and severe clinical types had elevated levels of PCT and NLR compared with moderate group (*P* < 0.05). Meanwhile, there was a statistical increased trend of CRP among the three groups, with the lowest in the moderate group (median 10 mg/L), then in the severe group (median 26.86 mg/L), and the highest in the critically severe group (median 92.2 mg/L) (*P* < 0.05), shown in Fig. 1.

### Diagnostic value of PCT, CRP and NLR for COVID-19 mortality

**Figure 2:**
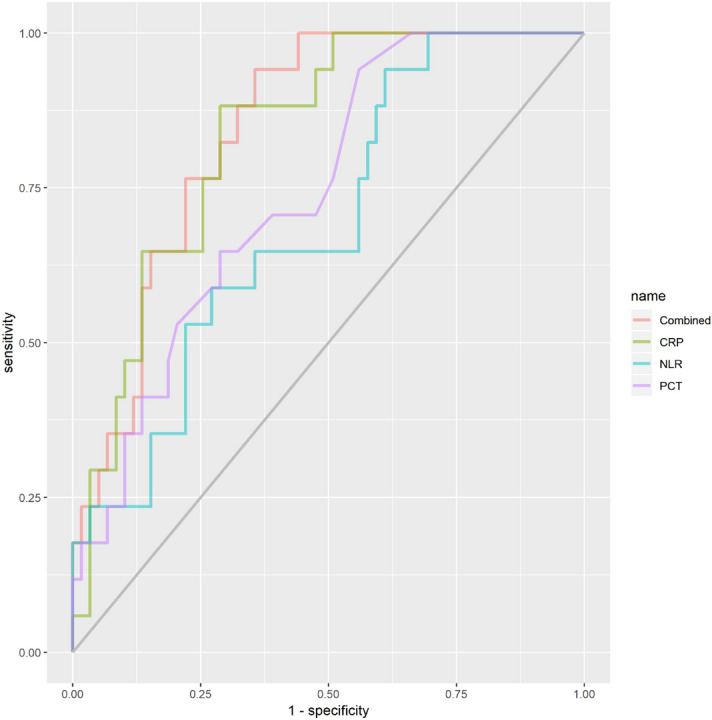
ROC analysis of the PCT, CRP, NLR and combined effect for prediction of COVID-19 mortality. The reference line was coloured with grey; the ROC of CRP was coloured with light-green; ROC of PCT was coloured with light-purple; ROC of NLR was coloured with light-blue; and ROC of combined PCT, CRP and NLR together was coloured with light-pink.

From the Fig. 2 and Table 2, the PCT, CRP, NLR and their combined effect had diagnostic values for COVID-19 mortality (*P* < 0.05), and the AUC from highest to lowest was combined effect > CRP > PCT > NLR, respectively. However, the diagnostic value for combined effect was not statistically superior to CRP with specificity of 0.64 and 0.71, respectively.

**Table 2:** The diagnostic value of PCT, CPR, NRT and combined effect for COVID-19 mortality.

Variables	AUC	95% *CI*	Cut-off value	Sensitivity	Specificity
PCT, ng/mL	0.74	0.62–0.87	0.10	0.94	0.44
CRP, mg/L	0.83	0.73–0.93	52.13	0.88	0.71
NLR	0.69	0.56–0.83	3.59	0.94	0.39
Combined (logit)	0.84	0.75–0.93	0.12	0.94	0.64

*COVID-19* coronavirus disease 2019, *AUC* area under the curve, *95%CI* confidence interval *PCT* procalcitonin, *CRP* C-reaction protein, *NLR* neutrophil-to-lymphocyte ratio.

### Risk factor for COVID-19 mortality using univariate analysis

**Figure 3:**
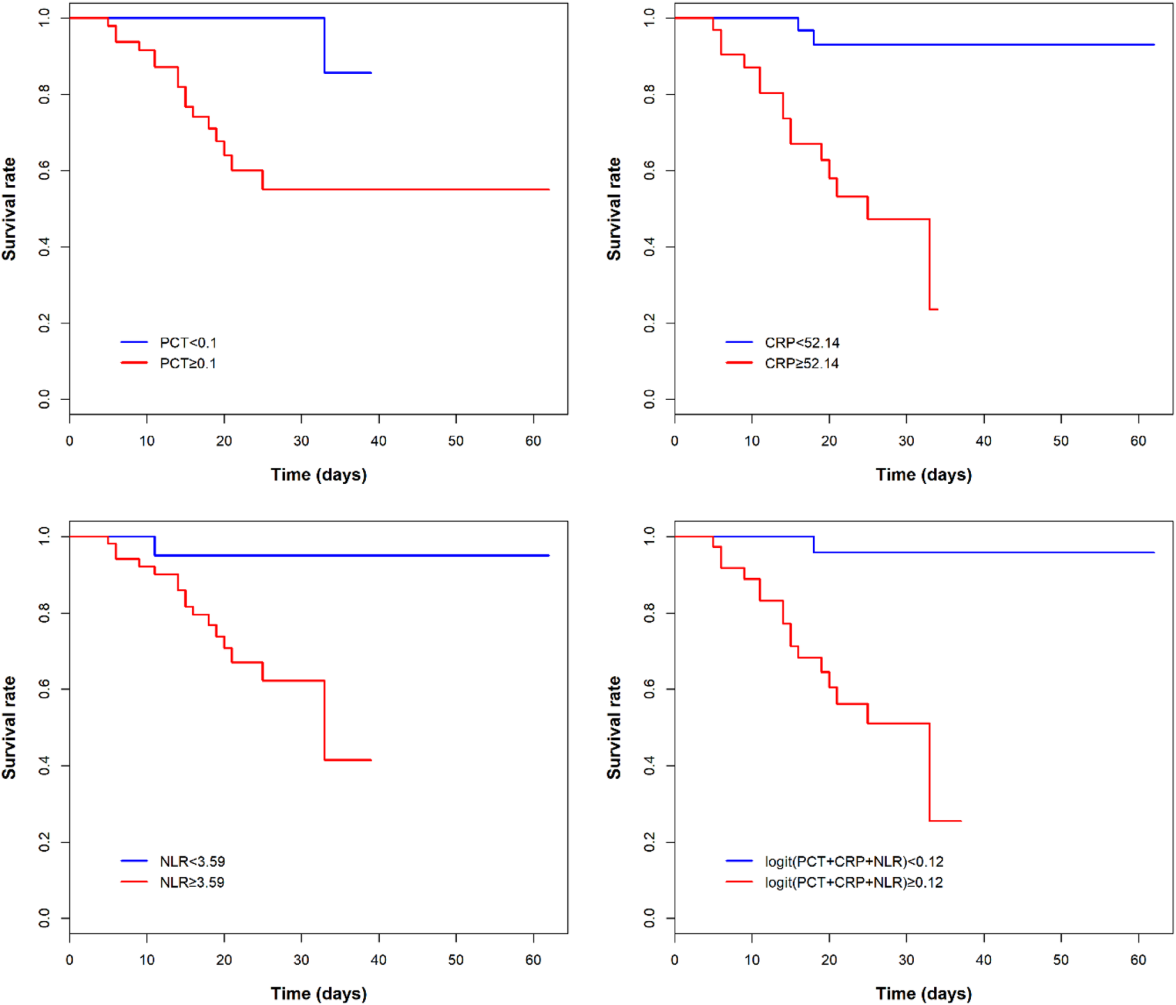
Kaplan–Meier plot for probability of survival for PCT, CRP, NLR and combined effect for prediction of COVID-19 mortality. The blue lines were the patients with COVID-19 with lower levels of inflammatory factors, and the red lines were the patients with COVID-19 with higher levels of inflammatory factors.

According to the cut-off-value of AUC, the levels of PCT, CRP and NLR were categorized into two groups. Results shown that several clinical factors were statistically significant risk associated with COVID-19 mortality, which included cancer, ARDS, hypohepatia, renal insufficiency, heart failure and shock. Furthermore, we found that PCT (≥ 0.10 ng/mL), CRP (≥ 52.14 mg/L) and NLR (NLR ≥ 3.59) had higher risks associated with higher likelihood of developing death with HRs of 12.82, 12.30 and 8.6, respectively (see Supplementary Table S1 online and Fig. 3).

### Risk factor for COVID-19 mortality using multivariate analysis

Adjustment for age and sex, the risk associations did not weaken between the PCT, CRP and NLR and death. Furthermore, PCT (≥ 0.10 ng/mL) and CRP (≥ 52.14 mg/L) exhibited independent increasing risks of mortality after adjusting for age, sex, cancer, ARDS, hypohepatia, renal insufficiency, heart failure and shock in model 2, with HRs of 52.68 (95% *CI*: 1.77–1571.66) and 5.47 (95% *CI*: 1.04–28.72), respectively. However, NRL (≥ 3.59) was not found to be an independent risk factor for death in multivariate analysis (Table 3).

**Table 3:** Risk factors between baseline PCT, CRP and NLR and death in patients with COVID-19 by using multivariate cox regression analysis.

Laboratory tests	Model 1^a^		Model 2^b^	
	*HR* (95% *CI*)	*P*	*HR* (95% *CI*)	*P*
PCT, ng/mL (≥ 0.10 vs. < 0.1)	**12.47 (1.63–95.32)**	**0.015**	**52.68 (1.77–1571.66)**	**0.022**
CRP, mg/L (≥ 52.14 vs. < 52.14)	**13.06 (2.95–57.86)**	**0.001**	**5.47 (1.04–28.72)**	**0.045**
NLR (≥ 3.59 vs. < 3.59)	**8.94 (1.13–70.59)**	**0.038**	0.82 (0.07–10.13)	0.875

Data in bold indicate values of statistical significance.

*COVID-19* coronavirus disease 2019, *HR* hazard ratio, *95% CI* confidence interval, *PCT* procalcitonin, *CRP* C-reaction protein, *NLR* neutrophil-to-lymphocyte ratio.

^a^Model 1 was adjusted for age and sex.

^b^Model 2 was adjusted for age, sex, cancer, ARDS, hypohepatia, renal insufficiency, heart failure and shock.

### Risk factor for COVID-19 mortality in the subgroup analysis

In the subgroup analysis (Table 4), we found the elevated PCT level was associated with increasing risk of mortality in the old age group (age ≥ 60 y), and the association did not change after adjusting for complications. In the severe group, a marginal association was found between PCT level and death (*HR* = 7.46, 95% *CI*: 0.98–56.54), however, the association increasing risk of mortality was observed after adjustment for complications (*HR* = 39.37, 95% *CI*: 1.50–1,036.84).

**Table 4:** Risk factors between baseline PCT, CRP and NLR and death in patients with COVID-19 in the subgroup analysis.

Laboratory tests	Characteristics	*n*	Model 0^a^		Model 1^b^	
			*HR* (95% *CI*)	*P*	*HR* (95% *CI*)	*P*
PCT, ng/mL (≥ 0.10 vs. < 0.1)	Age (< 60y)	37	3.76e+8 (0–Inf)	0.999	84,176.46 (0–Inf)	0.989
	**Age (≥ 60y)**	39	**10.08 (1.29**–**78.80)**	**0.028**	**53.76 (1.02**–**2,824.88)**	**0.049**
	Male	46	6.27 (0.79–49.89)	0.083	1.53e+6 (0–Inf)	0.989
	Female	30	1.28e+9 (0–Inf)	0.999	1.58 (0–Inf)	0.999
	Severe*	50	**7.46 (0.98**–**56.54)**	**0.052**	**39.37 (1.50**–**1,036.84)**	**0.028**
CRP, mg/L (≥ 52.14 vs. < 52.14)	Age (< 60y)	37	4.39 (0.48–39.73)	0.188	2.93e+13 (0–Inf)	0.990
	**Age (≥ 60y)**	39	**23.59 (3.01**–**185.03)**	**0.003**	14.95 (0.78–287.68)	0.073
	**Male**	46	**6.37 (1.29**–**31.41)**	**0.023**	2.29e+16 (0–Inf)	0.998
	Female	30	3.06e+9 (0–Inf)	0.999	8.17e+20 (0–Inf)	0.948
	Severe*	50	**5.54 (1.26**–**24.28)**	**0.023**	4.72 (0.91–24.56)	0.065
NLR (≥ 3.59 vs. < 3.59)	Age (< 60y)	37	3.38e+8 (0–Inf)	0.999	3.57e+8 (0–Inf)	0.998
	Age (≥ 60y)	39	5.92 (0.74–47.58)	0.094	0 (0–Inf)	0.960
	Male	46	3.56e+8 (0–Inf)	0.998	2.56e+8 (0–Inf)	0.999
	Female	30	3.38 (0.4–28.28)	0.261	0 (0–Inf)	0.960
	Severe*	50	0.81 (0.1–6.58)	0.841	3.47 (0.43–28.14)	0.244

Data in bold indicate values of statistical significance.

*COVID-19* coronavirus disease 2019, *HR* hazard ratio, *95% CI* confidence interval, *PCT* procalcitonin, *CRP* C-reaction protein, *NLR* neutrophil-to-lymphocyte ratio.

^a^Model 0 was unadjusted.

^b^Model 1 was adjusted for cancer, ARDS, hypohepatia, renal insufficiency, heart failure and shock.

*Severe included critical severe and severe clinical types.

We also found the elevated CRP levels were associated with increasing risk of mortality in the old age group (age ≥ 60 y), in male group and severe group. However, there were no risk associations after adjusting for complications (*P* > 0.05). Unfortunately, NLR level had not been addressed as independent predictor of survival in patients with COVID-19 in different age, sex and severe groups (*P* > 0.05).

## Discussion

Our results had demonstrated that patients in the critically severe and severe groups had elevated levels of PCT and NLR compared with moderate group, and the CRP level was highest in the critically severe group, the lowest in the moderate group as expected. After Adjusting for other covariates, the serum elevated PCT (≥ 0.10 ng/mL) and CRP (≥ 52.14 mg/L) were independent risk factors for mortality in hospitalized patients with COVID-19. However, elevated NLR (≥ 3.59) was not the independent risk factor for mortality in our study. Furthermore, the elevated PCT levels were still associated with increasing risk of mortality in the old age group (age ≥ 60 y) and in the critically severe and severe patients after adjusting for complications.

The reported observed symptoms of COVID-19 were generally non-specific, the fever was the most common symptom, followed by cough and fatigue, dyspnea and et al.^21^. In our study, the fever and the cough were also found as the common symptoms, followed the chest distress or shortness of breath. This may be because the patients with COVID-19 in our study were not included the mild clinical type. The overall case-fatality rate was 22.37% in our study, and all the deaths were observed in the critically severe group with mortality rate of 53.13% (17/32), which was consistently with previous research^22^.

COVID-19 is a highly infectious pneumonia caused by a SARS-CoV-2, and other sensitive indicators such as inflammation factors had to reflect lung lesion changes and disease severity except for CT scan^23^. PCT is a calcitonin-related gene product expressed by human epithelial cells in response to bacterial infections^24^, which is used as a blood infection biomarker for the purpose of guiding antibiotic therapy in the context of pulmonary infection^25,26^. CRP, an acute-phase protein secreted by the liver, is another parameter used to response to bacterial infection, which was used in the early diagnosis of pneumonia^27^.

In this study, our results had found that the levels of PCT and CRP were associated with COVID-19 severity, which indicated patients with COVID-19 always companied by the bacterial infections. Also, the serum levels of PCT and CRP had diagnostic values for COVID-19 mortality with higher sensitivity. After adjusting for other covariates, the serum elevated PCT (≥ 0.10 ng/mL) and CRP (≥ 52.14 mg/L) were independent risk factors for mortality in hospitalized patients with COVID-19. It’s worth noting that the death for old patients (age ≥ 60 y) and severe patients were more susceptible to elevated PCT. However, this association was not observed in the CRP biomarker after adjusting for complications. From the infinity *HR*s and marginal *P* value (0.065), we expected that small sample size may be the most reasons for this association. Thus, elevated levels of PCT and CRP may help to identify patients with dismal prognosis and prompt intervention in order to improve outcomes, especially in the old and severe patients with COVID-19.

NLR was an another important inflammation indicator, which was associated with a poor prognosis for patients with many disease^28,29^. The NLR reflects the balance between the innate (i.e., neutrophils) and adaptive (i.e., lymphocytes) immune responses in the body^30^, and the elevated NLR values were susceptible for progress of COVID-19 infection^11,31^. Researches had indicated that several factors may contribute to COVID-19 associated lymphopenia. Firstly, the angiotensin-converting enzyme 2 (ACE2) plays an important role in cellular entry for SARS-CoV-2, which invades host human cells by binding to ACE2 receptor, thus the ACE2-expressing cell lymphocytes act as target cell and are leaded to lysis by COVID-19 infection^32^. Secondly, COVID-19 was found to be characterized as a “cytokine storm” with a pronounced systemic increase of inflammatory mediators and cytokines^33^, and those increased concentrations of cytokines may promote lymphocyte apoptosis^34^. Furthermore, increased activation of cytokines may impair lymphocyte turnover by atrophy of lymphoid organs^35^ or inhibiting lymphocyte proliferation^36^.

Our results had demonstrated that critical and severe clinical patients had elevated NLR levels compared with moderate group. However, in this study, we did not find the elevated NLR (≥ 3.59) was associated with increased risk of death, neither in other subgroup population.

Furthermore, we also explored the associations between the highest levels of PCT, CRP and NLR and mortality in patients with COVID-19 during the treatment, however, no significant risk factors were observed. Meanwhile, we categorized PCT and CPR levels into different groups according to the normal reference values of less than 0.05 ng/mL and 3 mg/L, respectively, and no significant associations with increased risk of death were found.

Some limitations existed in the present study. First, the available sample size and number of deaths were limited, which may reduce the validity when building prediction model and increase the risk of overfitting the model. Second, the inflammation factors were not monitored regularly, thus we could not longitudinally evaluate the association between the dynamic changes of PCT, CRP and NLR and death during the disease course. Third, the identified cutoffs for PCT, CRP and NLR in this study need to be validated in external patients with COVID-19.

## Conclusions

In this study, we confirmed that baseline levels of PCT (≥ 0.10 ng/mL) and CRP (≥ 52.14 mg/L) have been addressed as independent predictors of survival in patients with COVID-19, but the elevated NLR do not seem useful for discriminating the death in COVID-19 infected patients. Furthermore, elevated PCT levels were still associated with increasing risk of mortality in the old age group (age ≥ 60 y) and in the critically severe and severe patients after adjustment for complications. Therefore, early laboratory indices at baseline can assist clinicians in formulating a tailored treatment approach and promptly provide intensive care to those who are in greater need. However, small sample size of patients is short of representive, in the future, the researchers will collaborate in the world with a few medical facilities in the area to confirm the relationship between the inflammation factors and survival in patients with COVID-19 to improve the reliability of the study.

## Supplementary information

Supplementary Information.

## Data Availability

The datasets generated during and/or analysed during the current study are available from the corresponding author on reasonable request.
