# Supplementary material for: Associations of procalcitonin, C-reaction protein and neutrophil-to-lymphocyte ratio with mortality in hospitalized COVID-19 patients in China
Source: Sci Rep. 2020 Sep 14;10:15058. doi: 10.1038/s41598-020-72164-7 (PMC7490259; doi:10.1038/s41598-020-72164-7)
Supplement: Supplementary file 1 — Supplementary Information. [file 41598_2020_72164_MOESM1_ESM.pdf]

Associations of procalcitonin, C-reaction protein and Neutrophil-to-Lymphocyte Ratio with mortality in hospitalized COVID-19 patients in China

Jian-bo Xu [1†], Chao Xu [2†], Ru-bing Zhang [3], Meng Wu [2], Chang-kun Pan [4], Xiu-jie Li [5], Qian Wang [6], Fang-fang Zeng [7*], Sui Zhu [6*]

[1] Department of Critical Care Medicine, School of Clinical Medicine, Jiamusi University, No.348, Dexiang Street, Jiamusi 154000, Heilongjiang Province, China.

[2] Department of Neurology and Respirator Intensive Care Unit, Heilongjiang Provincial Hospital, No. 82, Zhongshan Road, Harbin 150036, Heilongjiang Province, China.

[3] Department of Cardiology, School of Clinical Medicine, Jiamusi University, No.348, Dexiang Street, Jiamusi 154000, Heilongjiang Province, China.

[4] Department of Critical Care Medicine, Jiamusi Tumor Hospital, No.37, Guanghua Road, Jiamusi 154000, Heilongjiang Province, China.

[5] Department of Respiratory Medicine, Jiamusi Tumor Hospital, No.37, Guanghua Road, Jiamusi 154000, Heilongjiang Province, China.

[6] Department of Medical Statistics, School of Medicine, Jinan University, No.601 Huangpu Road West, Guangzhou 510632, Guangdong Province, China.

[7] Department of Epidemiology, School of Medicine, Jinan University, No.601 Huangpu Road West, Guangzhou 510632, Guangdong Province, China.

Supplementary Table S1. Risk factors associated with mortality in patients with COVID-19 by using univariate Cox model analysis

Variables	Survival (n=59)	Death (n=17)	*HR*	95% *CI*	*P*
**Demographic**					
Age, mean (SD), years	57.41 (14.97)	65 (11.49)	1.03	0.99 - 1.07	0.149
Sex (male vs female)	23 (38.98)	7 (41.18)	1.14	0.43 - 3.01	0.786
Time from illness onset to first hospital admission, median (IQR), days	7 (8)	7 (6)	0.96	0.87 - 1.05	0.344
Chronic heart disease (yes vs no)	6 (10.17)	1 (5.88)	0.55	0.07 - 4.13	0.558
Chronic lung disease (yes vs no)	2 (3.39)	0 (0)	0	0 – Inf [&]	0.998
Chronic kidney disease (yes vs no)	4 (6.78)	1 (5.88)	0.96	0.13 - 7.27	0.970
Diabetes (yes vs no)	9 (15.25)	6 (35.29)	1.93	0.71 - 5.23	0.196
Hypertension (yes vs no)	19 (32.2)	8 (47.06)	1.60	0.62 - 4.18	0.333
Cancer (yes vs no)	**1 (1.69)**	**2 (11.76)**	**4.83**	**1.08 - 21.66**	**0.040**
**Clinical symptoms**					
Fever (yes vs no)	50 (84.75)	14 (82.35)	0.55	0.15 - 1.94	0.350
Cough (yes vs no)	37 (62.71)	12 (70.59)	1.3	0.45 - 3.73	0.626
Headache (yes vs no)	2 (3.39)	0 (0)	0	0 – Inf [&]	0.998
Myodynia (yes vs no)	1 (1.69)	1 (5.88)	3.65	0.48 - 27.97	0.213
Chills (yes vs no)	6 (10.17)	2 (11.76)	1.18	0.27 - 5.22	0.823
Nausea and vomiting (yes vs no)	1 (1.69)	1 (5.88)	2.70	0.36 - 20.57	0.337
Chest distress or shortness of breath (yes vs no)	24 (40.68)	10 (58.82)	2.20	0.83 - 5.82	0.113
**Complications**					
ARDS (yes vs no)	**9 (15.25)**	**16 (94.12)**	**39.05**	**5.16 - 295.25**	**<0.001**
Hypohepatia (yes vs no)	**7 (11.86)**	**6 (35.29)**	**2.86**	**1.04 - 7.87**	**0.042**
Renal insufficiency	**0 (0)**	**6 (35.29)**	**7.10**	**2.6 - 19.36**	**<0.001**

(yes vs no)					
Heart failure (yes vs no)	**2 (3.39)**	**5 (29.41)**	**4.77**	**1.64 - 13.85**	**0.004**
Shock (yes vs no)	**3 (5.08)**	**12 (70.59)**	**14.03**	**4.90 – 40.18**	**<0.001**
**Therapy**					
Glucocorticoid (yes vs no)	42 (71.19)	17 (100)	3.02e+8	0 - Inf[&]	0.998
Mechanical ventilation (yes vs no)	35 (59.32)	17 (100)	4.22e+8	0 - Inf[&]	0.997
Immune globulin (yes vs no)	34 (57.63)	14 (82.35)	3.37	0.95 - 11.96	0.060
**Laboratory tests**					
PCT, ng/mL (⩾ 0.10 vs < 0.1)	**0.11 (0.18)**	**0.25 (0.27)**	**12.82**	**1.68 - 97.8**	**0.014**
CRP, mg/L (⩾ 52.14 vs <52.14)	**16.94 (61.71)**	**109.58 (126.7)**	**12.30**	**2.79 - 54.19**	**0.001**
NLR (⩾ 3.59 vs < 3.59)	**5.41 (7.84)**	**10.72 (9.67)**	**8.6**	**1.11 - 66.82**	**0.040**

[&] due to the absence of death.

COVID-19: coronavirus disease 2019; **HR**: hazard ratio; **95%** ***CI***: confidence interval; **SD**: standard deviation; **IQR**: interquartile range; **ARDS**: acute respiratory distress syndrome; **PCT**: procalcitonin; **CRP**: C-reaction protein; **NLR**: neutrophil-to-lymphocyte ratio.
